# Combination high-dose interleukin-2 and nivolumab for programmed cell death-1 refractory metastatic melanoma: a case series

**DOI:** 10.1186/s13256-022-03536-y

**Published:** 2022-08-31

**Authors:** Mina Nikanjam, Jaren Mullen, Carol Yacoub, Gregory A. Daniels

**Affiliations:** 1 Division of Hematology-Oncology, University of California San Diego, 3855 Health Sciences Dr #0987, La Jolla, CA USA; 2 Moores Cancer Center Clinical Pharmacy, University of California San Diego, 3855 Health Sciences Dr #0987, La Jolla, CA USA

**Keywords:** Interleukin-2, Anti-PD-1, Metastatic melanoma, Case report

## Abstract

**Background:**

Therapeutic options are needed for metastatic melanoma refractory to therapies directed against programmed cell death-1. High-dose interleukin-2 has the potential to overcome programmed cell death-1 resistance.

**Case presentation:**

We report three consecutive Caucasian patients, two female (60 and 55 years old) and one male (56 years old), refractory to anti-programmed cell death-1 therapy who were treated with concurrent nivolumab and standard-dosing bolus high-dose interleukin-2. We did not see any unexpected toxicities with overlapping treatments as compared with either high-dose interleukin-2 or nivolumab alone.

**Conclusions:**

The tolerance and disease control observed among the three patients in this limited series support formal exploration of this combination.

## Background

Anti-PD-1-directed checkpoint inhibitors (pembrolizumab and nivolumab) are monoclonal antibodies against the surface receptor programmed cell death-1 (PD-1), approved for treatment of metastatic melanoma. Nivolumab in combination with ipilimumab, a cytotoxic T-lymphocyte associated protein 4 (CTLA-4) inhibitor, demonstrated superior response rates compared with either single agent as monotherapy and improved survival compared with ipilimumab single agent, but at a cost of greater rates of toxicities [1]. While response rates approach 60% with combination therapy, many patients will progress and be refractory to PD-1-based therapy [1].

Prior to checkpoint inhibitors, high-dose interleukin-2 (HD IL-2) demonstrated modest response rates in select patients, but more importantly showed durable responses from immunotherapy for melanoma [2]. Interleukin-2 (IL-2) is a cytokine produced by primed CD4 T helper cells, activated CD8 T cells, activated dendritic cells, and natural killer T cells [3]. IL-2 drives proliferation and activation of CD8 T cells and natural killer cells in addition to the proliferation and antibody secretion of B cells [3]. IL-2 can also act as an immunomodulator by expanding immunosuppressive CD4 T-regulatory cells and promoting activation-induced cell death of T cells [3].

A prior study explored response rates and toxicity of HD IL-2 following anti-PD-1 or PD-L1-directed therapy [4]. This study found best overall response rates of 23% and a toxicity profile similar to historical HD IL-2 treatments with a possible increased incidence of pneumonitis [4]. This case report describes three consecutive patients who previously progressed on PD-1 pathway blockade and who were treated with HD IL-2 concurrent with anti-PD-1 directed therapy.

## Case presentation

### Patient 1

Patient 1 is a 60-year-old Caucasian female with BRAF wild type unknown primary (tumor mutation burden 4 mutations/megabase) metastatic melanoma to bone, lung, and soft tissue who originally presented with a renal mass and had a nephrectomy positive for melanoma. She developed lung disease and was started on pembrolizumab. After 3 months, her lung disease progressed, and she was switched to ipilimumab 3 mg/kg and nivolumab 1 mg/kg for four cycles with concurrent radiotherapy to a dominant lung lesion. Her maintenance nivolumab was halted after 2 months as she required steroids for radiation pneumonitis. She subsequently received anti-PD-1 directed therapy with anti-LAG-3 on a clinical trial, but her disease progressed after two cycles with progressive bilateral pulmonary and pleural based masses, increase in a right pericardial deposit, enlargement of a soft tissue mass adjacent to the spleen and a nodule adjacent to the right kidney with an unchanged lytic lesion within the L3 vertebral body.

She received 18 Gy in one fraction of palliative radiation to the L3 spinal lesion 3 days prior to HD IL-2 for pain (day 1). She was then admitted for HD IL-2 and received nine consecutive doses on days 4 through 7. Treatment was held for colitis and atrial fibrillation. She was administered nivolumab 480 mg on day 19. The patient was subsequently admitted for HD IL-2 and received nine consecutive doses on days 22 through 25. Therapy was held due to colitis and shortness of breath, with atrial fibrillation after dosing that resolved in a few days per usual after stopping HD IL-2.

CT scans on day 53 showed an 8% decrease in target lesions and overall stable disease per RECIST v1.1. The patient elected to receive a second course of nivolumab and HD IL-2 therapy with nivolumab 480 mg given on day 53 followed by standard bolus HD IL-2 on day 58 (7 doses) and day 77 (8 doses), both limited by fatigue. Usual side effects from HD IL-2-included colitis, renal insufficiency, shortness of breath, electrolyte issues, hepatitis, and mucositis all resolving to grade 1 or better at the time of dismissal from the hospital. She received two more doses of nivolumab 480 mg on schedule every 4 weeks with scans on day 73 and day 122 showing disease stability. Two months after her last dose of HD IL-2, she elected to change therapies to chemotherapy due to progressive pain. Scans on day 182, 6 months after the initiation of therapy, showed progression of disease due to growth of nontarget lesions. The patient experienced further disease progression, transitioned to hospice, and died on day 288.

### Patient 2

Patient 2 is a 56-year-old Caucasian male with NF1 mutated cutaneous metastatic melanoma (tumor mutation burden 1.3 mutations/megabase) who originally presented with a left finger stage IIIC melanoma. He was treated with surgery and sentinel lymph node biopsy. Scans showed extensive lymph node disease, and he was started on ipilimumab 3 mg/kg with nivolumab 1 mg/kg every 3 weeks, which was discontinued after three cycles due to hypophysitis ultimately requiring thyroid and adrenal hormone replacement. His cancer subsequently progressed, and he was started on a clinical trial of nivolumab with an anti-LAG-3 antibody, but after one cycle had clinical progression with extensive chest wall disease.

He was started on palliative radiation to the chest wall mass and received 45 Gy in ten fractions on days 1 through 11. He received 480 mg of nivolumab on day 6. He was admitted for HD IL-2 course 1 cycle 1 and received 11 doses on days 12 through 16. Treatment was held for thrombocytopenia and altered mental status. He also experienced atrial fibrillation with rapid ventricular rate, hyperbilirubinemia, colitis, and recurrent fevers after completion of HD IL-2 dosing, which resolved by discharge. He was admitted for cycle 2 of HD IL-2 and received 10 doses on days 33 through 36. Treatment was held for thrombocytopenia and altered mental status. Nivolumab 480 mg was administered on day 39.

**Fig. 1:**
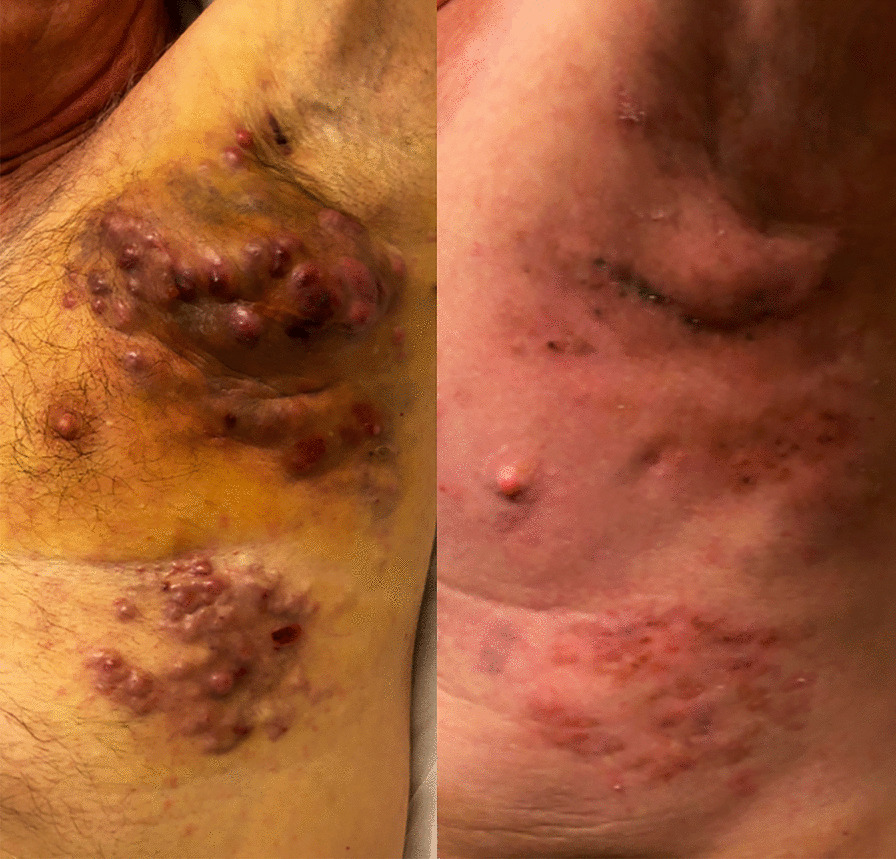
Digital photography of left chest wall disease for patient 2 before treatment (left) and after the first course of radiation and combination therapy (right), demonstrating dramatic improvement in skin disease

**Fig. 2:**
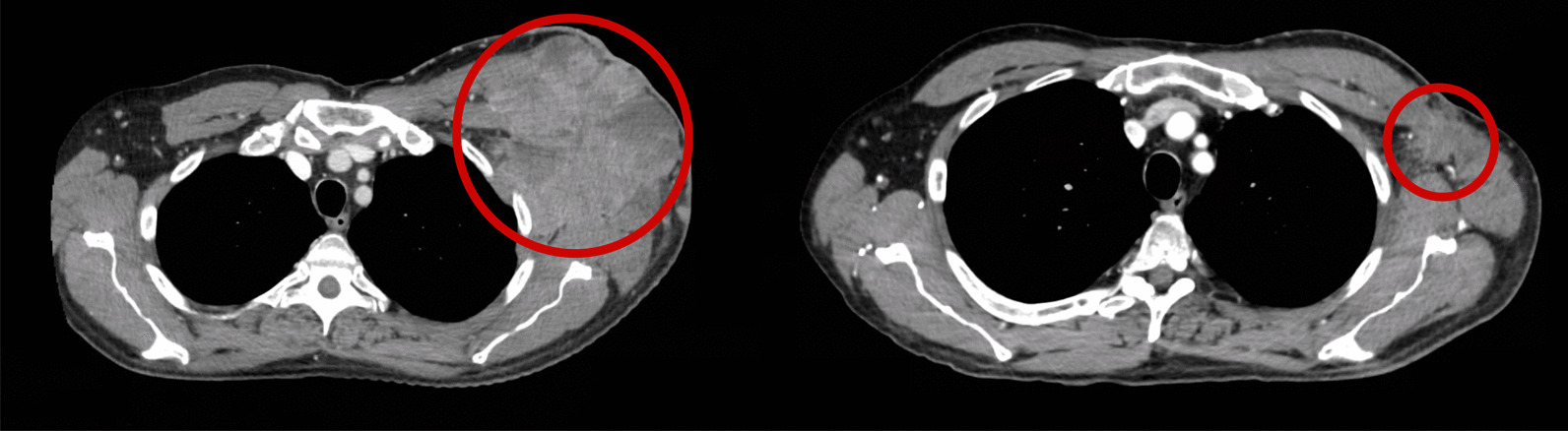
Axial computed tomography scan of chest for patient 2 before treatment (left) and axial computed tomography scan of patient 2’s chest after treatment with radiation, anti-PD-1, and high-dose IL-2 (right), demonstrating substantial reduction in tumor volume

Digital photography on day 72 (Fig. 1) and CT scans on day 71 (Fig. 2) showed dramatic improvement of disease in the chest wall and lungs (not irradiated). He had clinical improvement in a left upper arm lesion, which was not in the imaging field. The patient received further nivolumab on day 72 and was admitted for a second course of HD IL-2 on day 77 (10 doses). Treatment was again held for thrombocytopenia and altered mental status. He was admitted again on day 90 and received nine doses before therapy was held for altered mental status and abdominal pain. Nivolumab was administered on day 108, and subsequent scans on day 127 confirmed response to therapy with further decrease in target lesions. Scans on day 197 showed no evidence of disease. Follow-up scans on day 266, day 350, and day 454 also showed no evidence of disease. He later developed progressive anemia, and subsequent endoscopic evaluation on day 547 showed a bleeding mass in his stomach that was biopsy proven as metastatic melanoma. He elected to enroll into hospice and died on day 566.

### Patient 3

Patient 3 is a 55-year-old Caucasian female with BRAF wild type cutaneous metastatic melanoma (tumor mutation burden 4 mutations/megabase), aortic stenosis, and severe pulmonary hypertension. The patient had excisions of two melanomas 20 years prior, followed by adjuvant treatment with tamoxifen, cisplatin, IL-2, and IFNa. She had recurrent melanoma 2 years prior to presentation and received ipilimumab 3 mg/kg with nivolumab 1 mg/kg for four cycles followed by nivolumab maintenance with no clear benefit. She had talimogene laherparepvec injections, which were poorly tolerated, followed by resection, and an additional four doses of ipilimumab and nivolumab without benefit. The patient subsequently received arginase on a clinical trial with progression followed by a dose of carboplatin and paclitaxel for a progressive malignant pericardial effusion.

She received 39 Gy in 13 fractions of palliative radiation to her pelvic disease from day 1 until day 21. She had nivolumab 480 mg on day 19. The patient was admitted for HD IL-2 and received eight doses on days 22 through 25. Dosing was interrupted at dose number 4 due to fevers, rigors, and chest ache during red blood cell transfusion, possibly secondary to transfusion reaction. Therapy was resumed after 8 h and was stopped after her eighth dose due to worsening respiratory status with increased oxygen requirements and respiratory rate. The patient was dismissed 48 h after the last dose of HD IL-2 and symptoms resolved. The patient was readmitted for HD IL-2 and received nine consecutive doses from day 41 through 44. Therapy was held after dose 9 due to abdominal pain secondary to colitis and worsening respiratory status. The patient received nivolumab 480 mg on day 55.

CT scans on day 71 showed decreased tumor burden in bilateral pelvic masses, with the right pelvic mass being the primary radiation site. Formal response assessment for this patient is not possible due to the inclusion of measurable lesions in the radiation field. Further cycles were not given as she fractured her arm while working and subsequently chose to pursue other treatments. She later died on day 366.

## Discussion and conclusions

We describe three consecutive patients treated with overlapping standard dosing of anti-PD-1-directed therapy and HD IL-2. All patients had previously progressed on multiple prior treatments including anti-PD-1-directed therapies. None of the patients experienced unusual or excessive side effects from the combination therapy as compared with toxicities expected from HD IL-2 alone. The average number of doses of IL-2 reflects the toxicity and tolerability. Contemporary data in ipilimumab refractory patients found on average of nine doses for cycle 1 and eight doses for cycle 2 [5]. Despite these three patients’ multiple prior treatments and each with significant preexisting comorbidity (atrial fibrillation, adrenal insufficiency, and severe pulmonary hypertension), our patients experienced similar number of doses with cycle 1 (9, 11, 8) and cycle 2 (9, 10, 9). The number of doses is also consistent with our institutional average of ten doses in cycle 1 and nine doses in cycle 2 during course 1 of therapy (unpublished internal data). The dose-limiting toxicities were primarily cardiac, pulmonary, or neurologic, which also fits with our internal database of HD IL-2 administration. Thus, concurrent dosing of standard HD IL-2 with anti-PD-1 in heavily pretreated patients appears feasible at experienced centers.

There are many novel treatments for metastatic melanoma [6], but identifying additional new combination therapies is particularly important in checkpoint-resistant patients because approximately half of all metastatic melanoma patients progress on current standard treatments. A combination of anti-LAG-3 and PD-1 inhibition was recently approved based on improved progression-free survival data from NCT03470922, with objective response and overall survival data pending [7]. Response rates to HD IL-2 historically ranged from 15% to 23% with 5–10% complete responses [4, 5]. Patients resistant to PD-1 blockade appear to maintain the same historical response rate seen with HD IL-2 compared with therapy-naïve patients (roughly 20%) [4]. Several mechanisms may govern resistance to PD-1 inhibitors, including lack of an inflammatory response, activation of other checkpoints, loss of antigen expression, and the development of T cell exhaustion. IL-2 can address many of these barriers. In addition, IL-2 augments memory T-cell differentiation and responses and increases natural killer cells [3]. In mouse models, administering IL-2 in combination with anti-PD-L1 blockade showed synergistic increases in antigen-specific CD8 T cell numbers and function [8]. Targeting the PD-1 pathway has the potential to reverse T cell exhaustion, but is not always effective [9]. A recent randomized clinical trial (NCT03635983) utilizing a βγ-biased pegylated IL-2 formulation in combination with PD-1 inhibition compared with anti-PD-1 alone did not meet its primary endpoints, despite success with the combination in earlier phases [10]. It is possible that signaling through the α subunit of the IL-2 receptor is necessary to achieve a sufficient level of anticancer immune response. The possibility that the combination of anti-PD-1 and HD IL-2 may overcome resistance to anti-PD-1-directed therapy has prompted formal exploration in treatment-refractory metastatic melanoma and metastatic kidney cancer patients in NCT03991130.

## Data Availability

Data and source materials are available upon request.

## Abbreviations

HD IL-2: High-dose interleukin-2
PD-1: Programmed cell death-1
CTLA-4: Cytotoxic T-lymphocyte associated protein 4
IL-2: Interleukin-2
